# Supplementary material for: A protocol to develop a Quality Assessment Tool for Realist Synthesis (QUATRES)
Source: PLoS One. 2025 Sep 10;20(9):e0332250. doi: 10.1371/journal.pone.0332250 (PMC12422416; doi:10.1371/journal.pone.0332250)
Supplement: Attachment — Submitted filename: QUATRES Tool_reviewers response.docx [file pone.0332250.s003.docx]

**A protocol to develop** **A** **QUality Assessment Tool for REalist Synthesis (****QUATRES)**

Ferdinand C **Mukumbang^1^**; Brynne **Gilmore^2,3^**; Rebecca **Hunter^4^**; Sara **Dada^2,3^**; Sonia **Dalkin^5^**; Andrew **Booth^6,7^**

Dear Editors,

Thank for considering our manuscript for review and for the comments that the reviewer(s) have provided. We believe that these comments have helped us polish our manuscript towards potential publishing.

| **Reviewer comments** | **Authors’ response** |
| --- | --- |
| 1. Abstract – line 30 and Background line 58 – “A realist synthesis or review is a theory-driven, realist-informed interpretive approach to synthesizing secondary empirical data” Is the reference to ‘empirical’ data a bit misleading given that realist synthesis or review synthesize all types of data – not just empirical? | We agree with the reviewer. We have removed the word “empirical” so the sentence encompasses all data forms. |
| 2. Line114 “We will have to wait and see – need to close quotation brackets | Thank you for pointing this out. We have closed the bracket at the appropriate place. |
| 3. Line 338 and 332 refers to guidelines – will there be multiple guidelines? Or is this a typo? | Thank you. We have singularized the mentions of guidelines |
| 4. Line 339 – some typos identified here - ‘Ph.D. Realist Network and others are identified……  Should this be - PhD Realist Network and others as identified…… | Corrected. Thank you. |
| 5. Line 340 – should this sentence be the start of a new paragraph? “It is advised that….” And be linked to line 347 as part of the same paragraph? As it currently reads this section seems somewhat disjointed and needs to link more clearly.  For example, it is not clear how this sentence “endorsement of quality reporting guidelines should adopt specific language to avoid vagueness” links to the next bit about BioMed Central etc. | Thank you. Yes, this was meant to be a new paragraph and we have created a new paragraph with this sentence. We have streamlined this point by adding the following sentence:  In line with this practice, we plan to encourage the endorsement of QUATRES by appealing to journal Editors to refer authors to the QUATRES website and use the QUATRES guideline in tandem with RAMESES guidelines for their submitted manuscripts. |
| 6. Line 349 – Should this be “The Website will contain ….” | Yes, it should be “The Website will contain ….” |
| 7. Conclusion - this section seems rushed which is unfortunate as the conclusion is so important.  Line 384 – this first sentence in the conclusion seems out of place - is this about “Assessment of the quality of included studies? This is a component of the quality appraisal process but not what this protocol is about?  The following sentence seems to lose the focus of the protocol too – it starts off saying quality assessment is required “owing to the philosophical grounding and methodological flexibility of realist synthesis” – clear enough – but how will this “facilitate adoption of evidence generated from realist syntheses using a structured tool”?  The final sentence essentially says the same thing again but is not clear exactly how plans and steps to develop a quality assessment tool for the realist synthesis will support its adoption. | Thank you for this comment. We have reworked the conclusion to make it more robust.  The quality assessment of realist reviews is essential for informing the adoption of the evidence that emanate from them. Owing to the philosophical grounding and methodological flexibility of realist synthesis, we argue that a formal and comprehensive quality assessment is required to facilitate the adoption of evidence generated from realist syntheses using a structured tool. By linking the underlying philosophical tenets to the empirical practices involved in obtaining evidence in realist synthesis, QUATRES offers the potential to facilitate the translation of realist review findings into practice. This proposal outlines plans and steps to develop a quality assessment tool for the realist synthesis to support its adoption. |
| **Editorial requests** | |
| 1. Please ensure that your manuscript meets PLOS ONE's style requirements, including those for file naming. | We have done this. Thank you. |
| 2. Thank you for stating in your Funding Statement:  [This study was supported by the Health Research Board (Ireland) and the HSC Public Health Agency (Grant number ESI-2021-001) through Evidence Synthesis Ireland/Cochrane Ireland.].  Please provide an amended statement that declares *all* the funding or sources of support (whether external or internal to your organization) received during this study, as detailed online in our guide for authors at [http://journals.plos.org/plosone/s/submit-now](https://urldefense.com/v3/__http:/journals.plos.org/plosone/s/submit-now__;!!K-Hz7m0Vt54!kTLE-eLvpFdkfwoVUSv1RFx4xY8HA_n7OgcldfeltSHbqPfv5aQqPIzYJIWsoHI4i6fBpxaRWUWv8A$).  Please also include the statement “There was no additional external funding received for this study.” in your updated Funding Statement. | Thank you for the directive. We have added the statement “There was no additional external funding received for this study” to the funding statement. |
| Thank you for stating the following in your manuscript:  [This study was supported by the Health Research Board (Ireland) and the HSC Public Health Agency (Grant number ESI-2021-001) through Evidence Synthesis Ireland/Cochrane Ireland.]  We note that you have provided funding information that is currently declared in your Funding Statement. However, funding information should not appear in the Acknowledgments section or other areas of your manuscript. We will only publish funding information present in the Funding Statement section of the online submission form.  Please remove any funding-related text from the manuscript and let us know how you would like to update your Funding Statement. | I have removed the funding statement from the appendix section as requested. |
| 4. Thank you for stating the following in your Competing Interests section:  [NO authors have competing interests].  Please complete your Competing Interests on the online submission form to state any Competing Interests. If you have no competing interests, please state "The authors have declared that no competing interests exist.", as detailed online in our guide for authors at [http://journals.plos.org/plosone/s/submit-now](https://urldefense.com/v3/__http:/journals.plos.org/plosone/s/submit-now__;!!K-Hz7m0Vt54!kTLE-eLvpFdkfwoVUSv1RFx4xY8HA_n7OgcldfeltSHbqPfv5aQqPIzYJIWsoHI4i6fBpxaRWUWv8A$)  This information should be included in your cover letter; we will change the online submission form on your behalf. | Thank you. We have removed it. |
| 5. In your Data Availability statement, you have not specified where the minimal data set underlying the results described in your manuscript can be found. PLOS defines a study's minimal data set as the underlying data used to reach the conclusions drawn in the manuscript and any additional data required to replicate the reported study findings in their entirety. All PLOS journals require that the minimal data set be made fully available. For more information about our data policy, please see [http://journals.plos.org/plosone/s/data-availability](https://urldefense.com/v3/__http:/journals.plos.org/plosone/s/data-availability__;!!K-Hz7m0Vt54!kTLE-eLvpFdkfwoVUSv1RFx4xY8HA_n7OgcldfeltSHbqPfv5aQqPIzYJIWsoHI4i6fBpxb0GPY1KA$). | This is protocol paper and does not have any associate data |
| 6. Please provide a complete Data Availability Statement in the submission form, ensuring you include all necessary access information or a reason for why you are unable to make your data freely accessible. If your research concerns only data provided within your submission, please write "All data are in the manuscript and/or supporting information files" as your Data Availability Statement. | Thank you. We have done this. |
| 7. Please review your reference list to ensure that it is complete and correct. If you have cited papers that have been retracted, please include the rationale for doing so in the manuscript text, or remove these references and replace them with relevant current references. Any changes to the reference list should be mentioned in the rebuttal letter that accompanies your revised manuscript. If you need to cite a retracted article, indicate the article’s retracted status in the References list and also include a citation and full reference for the retraction notice. | Done |
