# Supplementary material for: A protocol to develop a Quality Assessment Tool for Realist Synthesis (QUATRES)
Source: PLoS One. 2025 Sep 10;20(9):e0332250. doi: 10.1371/journal.pone.0332250 (PMC12422416; doi:10.1371/journal.pone.0332250)
Supplement: Attachment — Submitted filename: I am happy to accept the manuscript.docx [file pone.0332250.s002.docx]

I am happy to accept the manuscript.

However, please note there is a linking word missing in the conclusion – line 389 - ‘is’ should be added as highlighted below in red text:

The quality assessment of realist reviews is essential
